# Teaching the placement of posterior resin-based composite restorations in Spanish dental schools

**DOI:** 10.4317/medoral.17656

**Published:** 2012-02-09

**Authors:** Raquel Castillo-de Oyagüe, Christopher Lynch, Robert McConnell, Nairn Wilson

**Affiliations:** 1 DDS, PhD, Associate Professor. Department of Buccofacial Prostheses, School of Dentistry, Complutense University of Madrid (U.C.M.); 2 BDS, PhD, MFD, RCSI, FDS (Rest Dent), Senior Lecturer/Consultant, Department of Adult Dental Health, School; 3 PhD, BDS, FFD RCSI, Emeritus Professor of Restorative Dentistry, University College Cork, Ireland; 4 PhD, MSc, BDS, FDS, DRD RCSEd, FHEA, Professor of Restorative Dentistry, King’s College London Dental Institute, London, UK

## Abstract

Objectives: In an area of esthetic dentistry such as posterior composites, in which new materials and techniques are being devolved continuously, it is important to confirm that dental students have a clear understanding of the basic principles of clinical application of this knowledge. Considering that the preparation of dental graduates in Spain may be of interest to competent dental authorities and employers with whom they can work worldwide, this study investigated the teaching of posterior composite restorations in Spanish dental schools. Study design: In late 2009⁄ early 2010, a questionnaire seeking information on the teaching of posterior composites was emailed to the professor responsible for teaching operative dentistry in each of the fifteen dental schools having complete undergraduate dental degree programs in Spain. Results: The response rate was 100%. Most investigated topics did not show noteworthy differences depending on whether the schools were public or private. Variations were found among Spanish dental schools in both the amount and content of the teaching programs concerning posterior composite restorations. Differences were recorded in the teaching of cavity design, contraindications to composite placement, indications for liners and bases, matrix and wedging techniques, composite and bonding systems, light curing and finishing procedures for composite restorations. More consistency was observed in teaching methods of moisture-control, indirect composites and amalgam bonding. Conclusions: As recommended in previously surveyed countries, efforts must be made to promote harmonization of dental curricula to make it easier for graduates to work elsewhere, and to ensure they meet the needs of their patients on entering independent practice.

** Key words:**Aesthetic dentistry, composite restoration, dental education, teaching program, undergraduate dental student.

## Introduction

The performance of posterior composite restorations has improved over the last decades due to continual advancements in bonding and composite technologies (1). Patients increasingly request its use instead of amalgam for the restoration of posterior teeth (2,3). Moreover, the placement of resin composite as a direct restorative material in occlusal and occlusoproximal cavities is supported by evidence presented in current literature (4-6).

Despite the recognized durability and quality of amalgam restorations, resin composite materials have been described as more advantageous than dental amalgam as regards their facilitation of minimally invasive dentistry, aesthetic properties, lack of mercury content, adaptation to rounded and reduced cavities, capability of reinforcing the remaining tooth-structure, and superior fracture resistance of the restored teeth (7-10). Even a clinical study revealed a slightly higher survival rate of posterior resin composites: 91.7% at five year and 82.2% at ten year follow-up evaluations, compared to amalgam’s 89.6% and 79.2% (6).

Nevertheless, pressures from dental practice and traditional views within the dental profession have created tension regarding the appropriateness between the teaching of amalgam and resin composite materials in dental schools for the restoration of posterior teeth (11). As dental students of 2010 will continue their practice into de mid 2050s, the teaching they receive on restorative dentistry in contemporary education will influence their approaches to treatment over many years to come (3,11-13). Studies on teaching of posterior composite resin restorations have been conducted in Europe (14); Ireland and the United Kingdom (UK) (12); Canada (15); the United States (US) (3); Brazil (16); Japan (17,18); and Iran (13); that demonstrate notable differences in the teaching programs within and between the surveyed countries.

In the UK, a vocational training period is mandatory for dental graduates. In Japan (18) dental graduates must pass the government’s national final qualifying examination before receiving their licenses. Conversely, in many other countries (e.g., Spain), graduates are immediately responsible for patient care including the placement of posterior composite restorations. Therefore, the preparation of graduates in Spain may be of interest to future employers, directors of postgraduate training or professional colleagues with whom they can work in the European Union (EU) and worldwide (19). Global teaching should be consistent to avoid confusion (20).

Taking into account the growing demand for esthetic restorations in dental practice (21), and the need to homogenize the teaching methods according to the ongoing trends towards internationalization (19), this study investigated the teaching of posterior composites to undergraduate dental students in Spain.

## Material and Methods

In late 2009⁄ early 2010, a questionnaire seeking information on the teaching of posterior composite restorations was emailed to the professor responsible for teaching operative dentistry in each of the fifteen dental schools having complete undergraduate dental degree programs in Spain. The questionnaire included nineteen closed-ended questions (where participants were given a number of possible responses to a statement and asked to identify the most appropriate one), and ten open-ended questions (where respondents were given space in which to write a textual answer to a statement) relating to the preclinical and clinical teaching of posterior composite restorations. Subjects investigated in the questionnaire included the following.

• Current and expected percentages of amalgam and composite restorations placed by students for occlusal and occlusoproximal cavities.

• Types of posterior composite resin restorations taught. The first technique taught at present and the one to be taught in five years (amalgam or composite).

• Teaching of various aspects relating to posterior composite restorations, such as principles of cavity design, contraindications, use of rubber dam and other methods of moisture control, use of liners and bases, matrix and wedging techniques, forms and commercial brands of composite resin and bonding systems, indications for flowable composite, curing light units, and techniques for finishing the restorations.

• Teaching of indirect composite restorations and amalgam bonding.

• Fees for posterior amalgam and composite restorations placed by students.

No questionnaire was eliminated from this study because all items were properly completed in each case. Information received was entered onto a Microsoft Excel® spreadsheet (Microsoft Office 2010; Washington, US). To further describe the findings, the schools were divided into public (11 schools) and private (4 schools).

Descriptive statistical data analysis was performed. Results were expressed in terms of mean, range, and percentages to standardize the statistical method; this method is followed in analogous studies performed in other countries (3,12-18) and facilitated the results’ comparisons. Confidentiality was maintained. The study conformed to relevant ethical expectations and requirements.

## Results

Fifteen completed questionnaires were returned (response rate = 100%). Questionnaires were completed by either the head of the department or equivalent or by a senior member of the teaching staff with specific responsibility for teaching posterior resin composites. Most investigated topics did not show noteworthy differences depending on whether the schools were public or private. Variations in both the amount and content of teaching programs concerning the placement of posterior composite restorations were recorded among Spanish dental schools, and classified in the following groups.

Types of posterior composite resin restorations taught. All schools reported that they taught the placement of composite resin in occlusal and two-surface occlusoproximal cavities in premolars and molars. The only dental school (7%) that did not teach composite resin placement in three-surface occlusoproximal cavities in premolars intends to introduce it within the next five years.

Preclinical and clinical teaching of posterior composite resin and amalgam. Thirteen dental schools (87%) – being ten public (91%) and three private (75%) –, reported that they taught the preparation of posterior cavities for amalgam before teaching preparations for resin composite. Three public dental schools (27%) and all private anticipated that in five years they would be teaching posterior cavity preparations for composite resin before teaching those for amalgam. On average, 26.4% (range = 0 – 50%) of posterior restorations placed by undergraduate students were amalgam, whereas 43.5% were composite resin (range = 10 – 80%).

Respondents anticipated that in five years, 11% of posterior restorations placed by undergraduate students would be amalgam (range = 0 – 20%), whereas the majority of the remaining restorations (58.5%) would be composite resin (range = 10 – 100%).

Differences in cavity preparation. In contrast to the cavity design for posterior amalgam, for composite restorations, thirteen schools (87%) taught the preparation of rounded internal line angles; eleven schools (73%) taught bevelled proximal box margins; eleven schools (73%) taught ‘slot-type’ cavities (i.e., no occlusal component); ten schools (67%) taught no ‘extension for prevention’; and nine schools (60%) taught bevelling of occlusal cavosurface margins.

Among public schools, one (9%) taught its students to protect the cusps from occlusal contacts. Two schools (18%) insisted on enamel-tissue preservation. Another school (9%) reported that their students were told only to ‘remove the caries and flatten the tooth surfaces before placing a composite restoration’.

Contraindications. Contraindications to the placement of posterior composite restorations in Spanish dental schools are displayed in  Table 1. The most common contraindications taught were history of allergy to composite resin materials (thirteen schools: 87%); inability to place the rubber dam prior to restore occlusoproximal cavities (nine schools: 60%); presence of subgingival margins (nine schools: 60%); replacement of large amalgam restorations and susceptibility to caries in case of occlusoproximal cavities in molars (five schools: 33%); and buccolingual width of the occlusal portion exceeding two-thirds of the intercuspal width when restoring occlusoproximal cavities in premolar teeth (five public schools: 45%).

**Table 1:**
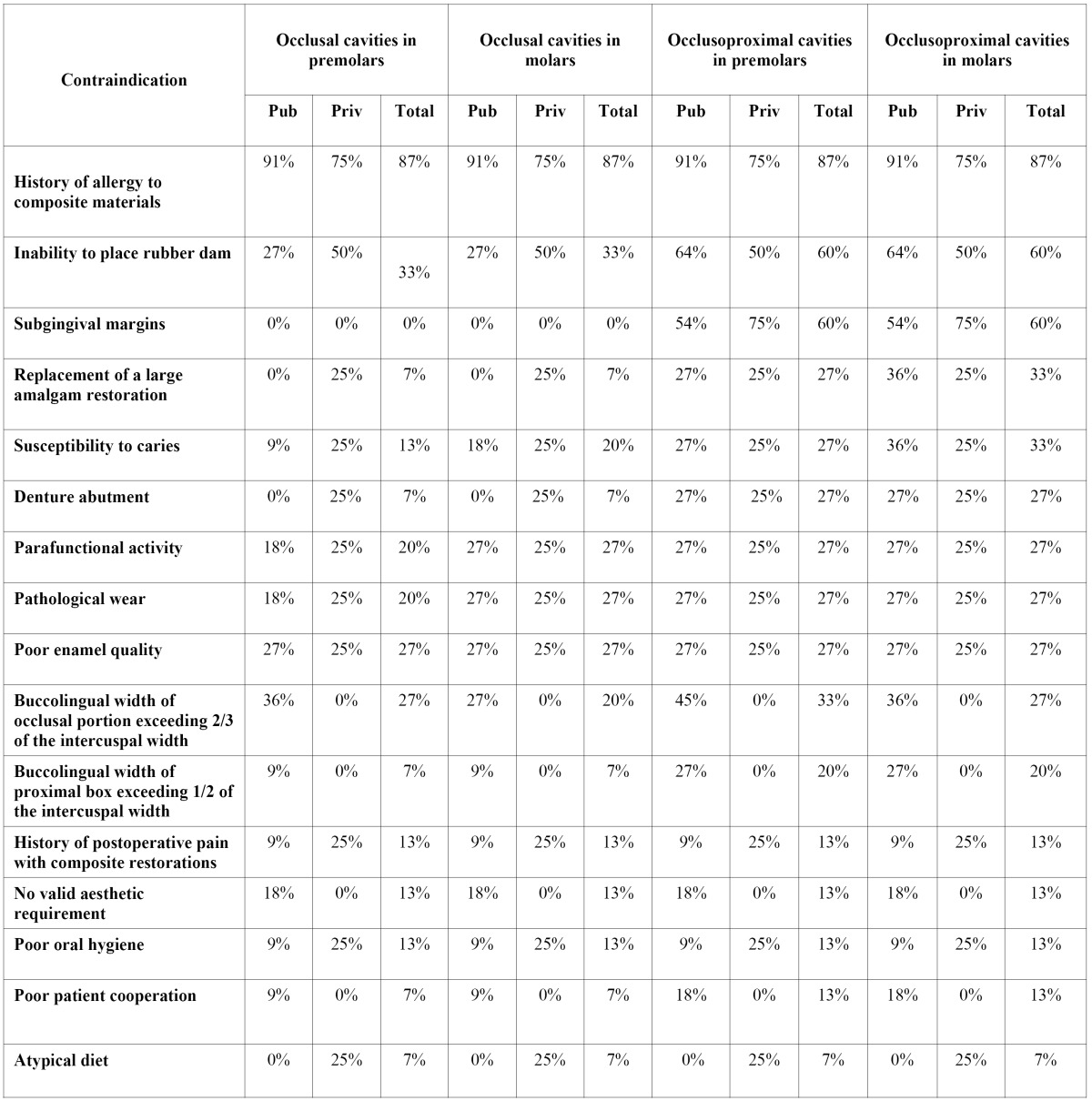
Percentages of Spanish dental schools teaching different contraindications to the placement of composite resin in posterior teeth.

Moisture control. Thirteen surveyed schools (87%) – nine public (82%) and all private – instructed their students in the use of rubber dam in most cases (> 75%) where posterior cavities were to be restored with composite resin. Alternative forms of moisture control included cotton wool rolls (eleven schools: 73%), and gauzes and dry guards (two public schools: 18%).

Two public (18%) and two private dental schools (50%) taught that there is no alternative to rubber dam to keep moisture under control in the placement of composite restorations.

Protection of operatively exposed dentine. The reported teaching on the use of liners and bases to protect operatively exposed dentine under posterior composite restorations is shown in  Table 2. The ‘total etch’ procedure, without the placement of any base or liner under the composite resin, was taught in thirteen schools (87%) for restoring shallow, and moderate cavities (i.e., located at the outer and middle third of dentine, respectively). This technique was taught for deep cavities (i.e., those in the inner third of dentine) in seven schools (47%), of which six were public (54%) and one private (25%). Conversely, in deep cavities, six other schools (40%) reported that their students were taught the use of glass ionomer cement without calcium hydroxide to seal the cavity. The remaining two public schools (18%) taught the use of calcium hydroxide plus glass ionomer cement.

**Table 2:**
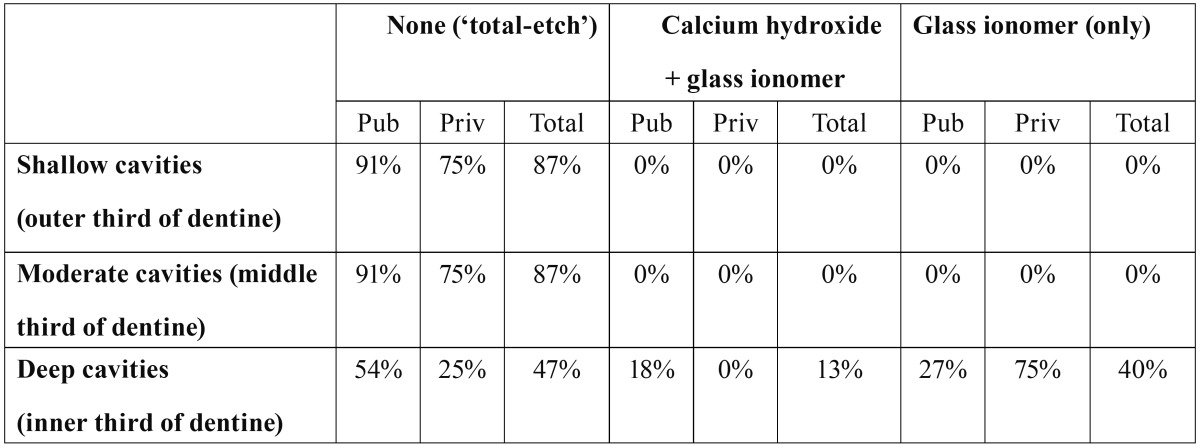
Percentages of Spanish dental schools teaching the use of liners and bases for posterior composite resin restorations.a

Matrix and wedging techniques. Seven schools (47%) taught only the use of a circumferential or sectional metal band in combination with a wooden wedge for occlusoproximal composite restorations. The remaining eight dental schools (53%) also taught the use of a transparent matrix band in association with a light transmitting wedge. Other matrix systems taught in public dental schools were auto-matrix rings (two schools: 18%); and customized matrices fixed with compound impression material or silicon moulds obtained from either wax patterns or study master casts (one school: 9%).

Commercial brands of composite resin and bonding systems taught. Five schools (33%) taught the use of hybrid composite; and four schools (27%) taught the use of nanofilled and/or nanofilled hybrid composite. The remaining six schools (40%) taught the use of hybrid, nanofilled and nanofilled hybrid composite resins for the restoration of posterior cavities so that a wide variety of commercial brands (up to fourteen) were used. The most common trademarks of composite resin materials introduced in teaching were ‘Tetric®’ (Ivoclar Vivadent; Schaän, Liechtenstein) (seven schools: 47%); ‘Esthet-Ex®’ (Dentsply/De Trey; Konstanz, Germany) (four schools: 27%); ‘Filtek®’ (3M ESPE; Seefeld, Germany) (three schools: 20%); ‘Grandio®’ (Voco; Cuxhaven, Germany) (three schools: 20%); and ‘Spectrum®’ (Dentsply/De Trey; Konstanz, Germany) (two schools: 13%). An average of 2.5 (min = 1; max = 8 in a public dental school) different brands of resin compo-site were taught per school.

Up to thirteen brands of total etch and self-etch bonding systems were taught in Spanish dental schools. The most common ones were: ‘Excite®’ (Ivoclar Vivadent; Schaän, Liechtenstein) (six schools: 40%); ‘Optibond Solo®/Optibond Solo Plus®’ (Kerr; Orange, CA, USA) (five schools: 33%); and ‘Prime & Bond NT®’ (Dentsply/De Trey; Konstanz, Germany) (four schools: 27%). An average of 2.21 (min = 1; max = 10 in a public dental school) different brands of bonding systems were taught per dental school.

Flowable composite. Five dental schools (33%) reported that they taught the use of flowable resin composite for restoring occlusal and/or occlusoproximal cavities. Three respondents (20%) recommended that their students use flowable resin composite exclusively in small occlusal cavities without occlusal contact. Ten dental schools (67%) taught the use of flowable composites as liners in deep occlusal cavities showing undercuts and irregularities. Two of these (13% of the surveyed schools) also taught the use of flowable resin composite as gingival liner in occlusoproximal cavities. Two schools (13%) taught the use of flowable composites as liners in gingival surfaces of proximal restorations.

Curing lights. Seven schools (47%) – four public (36%) and three private (75%) –, taught the use of ‘traditional’ quartz-halogen lamps for curing posterior composite resin restorations. Four public schools (36%) taught the use of light-emitting-diode (LED) curing lamps. Four schools (27%) taught the use of both light-curing systems. No school taught the use of plasma-arc lamps.

Finishing techniques. Fourteen schools (93%) taught ‘immediate’ finishing of posterior composite restorations, whereas only one (7%) taught ‘delayed’ finishing (after 24 h). Nine schools (60%) taught the use of water-cooling while finishing. Popular finishing instruments include diamonds and discs (twelve schools: 80%); stones (nine schools: 60%); strips (seven schools: 47%); and finishing pastes (six schools: 40%).

Indirect composite resin restorations. All schools reported that they taught techniques for indirect composite resin restorations. Nine schools (60%) reported that this teaching was theoretical only, but three schools (20%) said that their students also placed indirect restorations in phantom heads. The students of three schools (20%) gained clinical experience in this technique.

Amalgam bonding. All schools informed that they taught amalgam bonding. The most popular materials introduced for this purpose were ‘Panavia®/Panavia F®’ (Kuraray; Tokyo, Japan) (six schools: 40%); ‘Scotchbond 1®/Schotchbond Multipurpose®’ (3M ESPE; Seefeld, Germany) (three schools: 20%); and ‘Optibond Solo Plus®/Optibond FL®’ (Kerr; Orange, CA, USA) (two schools: 13%). In nine dental schools (60%), amalgam bonding was taught by means of theoretical lectures whereas in the remaining six schools (40%), didactic and clinical placement of amalgam bonding took place at least in the preclinical course.

Fee charges. Public and private dental schools in Spain charged fees for posterior amalgam and composite restorations placed by students, ranging from €20 to €50, depending on the extension of the cavity rather than on the type of restorative material.

## Discussion

Spain is an EU member state with 45,061,275 inhabitants and a total land area of about 505,954 km2. The number of dentists working in this country has increased by 102% in the last 15 years, from 13,242 dentists in 1994 to 26,725 in 2009, the majority of whom work in the private sector (data from the Spanish National Institute of Statistics: INE). An average of 1,060 dentists graduates from Spanish dental schools per year (645 from public and 415 from private schools).

Posterior composite resin restorations are an established feature of current dental practice (6,21). Therefore, a growing onus of responsibility falls on dental educators to ensure that students are adequately instructed in this restorative technique (3,11). Notwithstanding the evolution reported by Spanish dental schools, in which most posterior restorations placed by students are of composite resin, the present study detected variations in the teaching of relevant aspects such as principles of cavity design, contraindications, use of liners and bases, matrix and wedging techniques, commercial brands of composite resin and bonding systems, light curing technologies, and finishing methods for posterior resin composites. Such diversities must be audited, because they may affect the overall quality of restorations in general practice (17).

The present study indicates that the clinical teaching of posterior composites in Spain is comparable to that of described in other countries. 43.5% of posterior restorations placed by Spanish undergraduate students were composite. This compares to 42% of posterior restorations placed by undergraduate students in Iran (13); 45% in Japan (18); and 49% in Canada (15). Lower percentages of composite restorations were reported in the British Isles (12) and North America (30%) (3). Such differences probably depend on a number of factors, but may relate to the fact that the General Dental Service regulations in the UK do not include provision for occlusal and occlusoproximal composite restorations in premolar teeth (12); and to the strong emphasis still placed on silver amalgam in the US National Board examinations (3,11).

Dental schools in Spain estimated that the preclinical teaching of posterior composite restorations during the next five years will increase to 134% with regard to number of hours, while the preclinical teaching of amalgam restorations is set to decline by one-half its current level. As in other surveyed countries (12-15,17,18), these figures are to be welcomed, indicating that Spanish dental students gain internationally favorable, and rising levels of exposure to adhesive resin-based composite dentistry (13).

Before 2015, a change in trends of teaching programs of restorative dentistry is likely to be produced in three public schools (27%) and all private, that plan to teach posterior cavity preparations for composite resin before teaching those for amalgam. In the meantime, 73% of public schools will continue teaching amalgam as the first step. Studies should be made to identify the best teaching strategy to start clinical practice.

As for cavity design, thin composite layers bonded to bevelled occlusal cavosurface margins may fracture even under functional loads, which contraindicates this procedure (22). Although no school taught this type of occlusal preparation in Canada (15), Iran (13), and Japan (18), it is a matter of concern that 60% of Spanish schools still taught bevelling occlusal margins for composite restorations, duplicating the percentages of schools teaching this method in Ireland and the UK (12), and the US (3).

According to that recorded in other surveyed countries (3,12-18), the most common contraindication for placement of posterior composite restorations was history of allergy to resin-based materials ( Table 1). However, because of the low documented frequency of such hypersensitivity (23), this response from schools may reflect medico-legal concerns rather than instruction based on documented evidence (3).

It is also surprising that Class II premolar cavities, with an occlusal portion exceeding two-thirds of the intercuspal width, were not considered as a contraindication by 55% of the public and any of the private Spanish dental schools. This contraindication was not recognized in 24% of schools in Brazil (16); 30% of schools in Canada (15), and the British Isles (12); 25%-50% in the US (3); 50% in Japan (18); and 83% in Iran (13). This is alarming as polymerization shrinkage may affect the marginal seal (24), increasing the risk of microleakage and recurrent caries. Thus, an indirect restoration should be preferred when the possibility of cusp deflection following bulk polymerization is expected (16). Nevertheless, only 20% of Spanish dental schools procured clinical experience in the placement of indirect restorations, which was similar to that observed in Ireland and the UK (27%) (12) and Canada (30%) (15). Even though the teaching of indirect restorations should be addressed in all schools, these data improves the findings of past surveys in Europe (14), and Iran (13), where dental students where not trained in the placement of indirect composite restorations.

A lack of uniformity was also noted in the management of deep cavities. 47% of the schools taught the ‘total etch’ technique, and the remaining 53% taught the placement of bases or liners ( Table 2). This was also found to be the case in Canada (15), and Ireland and the UK (12); where the percentages of schools teaching the total etch technique for deep cavities were 20%, and 30%, respectively. Such inconsistency reflects the absence of consensus in the research community concerning the treatment of operatively-exposed dentin (1,15).

It is an excellent outcome that all schools taught the use of a circumferential or sectional metal band in association with a wooden wedge for placement of occlusoproximal composite resin restorations. This occurred as well in other countries (3,12,13,15,16,18); more than 90% of schools teach this technique. The better performance of such a method is supported by evidence in the literature (25). Resin composites provide little internal force to counter the force applied by the matrix band, unlike amalgams, which are more resistant to deformation (26). Hence, non-rigid matrices (e.g., transparent) and stiff wedges (e.g., light-transmitting ones), complicate achieving proper contact tightness and usually determine the formation of proximal overhangs because of worse adaptation (25).

It is also positive that in most schools a variety of newly-introduced materials and technologies are taught. Consistent with other countries (3,12,13,15,16,18), all schools taught the use of hybrid and microhybrid resin composite. Familiarity with several brands of composites and bonding systems encourages students to compare the quality of different products, increases their experience, and enables them to leave the dental school without being wedded to a specific restoring system (3).

More than 50% of Spanish schools taught the use of LED curing lights. This is in keeping with available best evidence, which suggests that the long-term degree of polymerization of composite reached with LED lights is comparable to that attained with traditional quartz-halogen units (27). Only Japanese schools (18) surpass this percentage, with 61% of the schools teaching the use of LED lights.

More questionable is that 90% of the schools taught immediate finishing of posterior composite restorations, given that delayed finishing has been recommended to release the stress of polymerization shrinkage generated at the resin-tooth interface by the hygroscopic expansion of resin composites (registered in the first 24 h or later) (17). However, this finding is in agreement with those from other countries –51% of Brazilian schools (16); 80% of European schools (14); 87% of British schools (12); and all surveyed schools in Canada (15), the US (3), and Iran (13), taught immediate finishing.

Higher consistency was observed in the teaching of moisture-control techniques, indirect composites (as described), amalgam bonding (taught in all schools likewise in most countries), and fees for posterior restorations. As in past surveys (3,12,13,15,16,18), the rubber dam was the preferred form of isolation. Resin-based materials are hydrophobic and highly technique sensitive. Therefore, apart from having other patient safety implications, the rubber dam becomes crucial to avoid contamination for a successful bonding (16,28). Regrettably, the presence of subgingival margins, where rubber dam cannot be placed, was not considered as a contraindication by 40% of the schools, which, along with the patterns observed in other countries (3,12,13,15,16,18), seemed to be incongruous. Finally, prices of posterior composite restorations placed by students in Spanish dental schools were comparable to those in Canada (15), and the US (3). Except some schools in Brazil (16), and Japan (17), all schools of other surveyed countries charged for composite restorations.

In light of the diversity of teaching observed among different countries, it is essential that dental undergraduate programs continually evaluate their portion of the restorative curricula to verify that the current therapeutic and technical goals related to the dental health, functional and aesthetic needs of society are being met. This study may be considered the first step in acquiring in-depth information to assess the evolution of the teaching of posterior composite restorations in Spanish dental schools. As recommended in previously surveyed countries (3,12-18), efforts must be made to promote harmonization of dental curricula among Spanish dental schools. This will make it easier for Spanish graduates to work elsewhere, and to ensure they meet the requirements of their patients on entering independent practice.
